# Cardiovascular Outcomes with Empagliflozin – News for Type 2 Diabetes Therapy

**DOI:** 10.17925/EE.2016.12.01.31

**Published:** 2016-03-15

**Authors:** Baptist Gallwitz

**Affiliations:** Dept Medicine IV, Eberhard Karls University, Tübingen, Germany

**Keywords:** Type 2 diabetes, SGLT-2 inhibitors, empagliflozin, cardiovascular outcomes

## Abstract

Cardiovascular safety has to be proven for antidiabetic therapy for type 2 diabetes according to the guidance from regulatory bodies. Recently, the results of the respective study for the sodium-glucose transporter-2 (SGLT-2) inhibitor empagliflozin were published. Unlike similar studies for other antidiabetic agents that proved cardiovascular safety by non-inferiority compared with standard treatment with regard to a combined cardiovascular primary endpoint, empagliflozin showed superiority with a significantly lower incidence of cardiovascular events.

Type 2 diabetes is a progressive chronic disease leading to vascular complications over the years when treated insufficiently.^1,2^ Treatment consists of a lifestyle intervention aimed at improving physical activity and healthy nutrition, later followed by oral antidiabetic drugs and injectable medications (glucagon-like peptide [GLP]-1 receptor agonists or insulin).^3,4^ Novel antidiabetic agents have had to prove cardiovascular safety since 2008, when guidance for mandatory specific cardiovascular safety studies was implemented by the United States Food and Drug Administration (FDA).

Sodium-glucose transporter-2 (SGLT-2) inhibitors are a novel class of antidiabetic agents that were introduced in 2012. SGLTs are cell membrane proteins that facilitate active glucose transport through the cell membrane. SGLT-2, a kidney specific isoform, is expressed in the renal tubule and is responsible for the physiological reabsorption of glucose from the proximal tubule after glomerular filtration. SGLT-2 inhibition therefore leads to removal of excess glucose via the urine in type 2 diabetes and improves glycaemia without an increased hypoglycaemia risk. SGLT-2 inhibitors have additional actions that may be favourable in type 2 diabetes: due to the glucosuria, a caloric loss occurs and in clinical studies a moderate loss of body weight between 2–3 kg is observed with SGLT-2 inhibitor treatment. Furthermore, blood pressure is reduced by approximately 2–5 mmHg together with slight volume and sodium depletion.^5^

A cardiovascular safety study according to the FDA guidance with the SGLT-2 inhibitor empagliflozin has now been published that not only proved cardiovascular safety but also reported a reduced risk of total mortality and a decreased risk of cardiovascular death in patients with type 2 diabetes at specifically high risk of cardiovascular events.^6^ Almost 50% of the patients enrolled in this study, BI 10773 (Empagliflozin) Cardiovascular Outcome Event Trial in Type 2 Diabetes Mellitus Patients (EMPA-REG OUTCOME) trial, had a previous history of myocardial infarction, in almost 75% coronary artery disease had been diagnosed and up to 25% had suffered from a stroke or had signs of peripheral vascular disease. The diabetes duration in the study cohort was also long, with a majority of patients being diabetic for more than 10 years. Likewise, signs of renal complications were also prevalent (microalbuminuria in approximately 30%; macroalbuminuria in 10% of patients). The majority of patients were on a combination therapy for their type 2 diabetes with antihyperglycaemic medications. Concomitant medications for hypertension and dyslipidaemia were widely used with >90% of patients receiving antihypertensive agents and 80% receiving statins or other lipid-lowering drugs.

Empagliflozin was added to standard treatment at two doses of either 10 or 25 mg once daily in the intervention group. In the comparator arm, standard care was intensified to reach comparable glycaemic outcomes in all study arms. Both empagliflozin doses resulted in a significant reduction of total mortality and the three-point major adverse cardiac events (MACE) rate, mainly driven by a reduction of cardiovascular death. The number needed to treat (NNT) was 39 over a time period of 3 years. Empagliflozin was well tolerated with an increased rate of genital infection as an adverse event (6.4% versus 1.8%) but no increase in other adverse events.^6^

The reported risk reduction was most likely multifactorial and not related only to glycaemic effects, since the difference in event rates occurred early during the study and glycaemic control was comparable in all study groups. SGLT-2 inhibitors may elicit vascular effects and may change renal as well as cardiac regulatory functions in water, electrolyte and blood pressure regulation. Albuminuria and uric acid as established surrogate parameters for cardiovascular risk were also reduced in the empagliflozin-treated patients. Other still unknown potentially beneficial effects of SGLT-2 inhibitors may also contribute to the study results.

Do these findings now make it necessary to change diabetes treatment and guidelines immediately? Certainly, studies showing a significant beneficial effect on cardiovascular outcomes with antidiabetic agents are scarce and comparable studies with dipeptidyl-peptidase-4 inhibitors (DPP-4 inhibitors) have only shown non-inferiority versus standard care, but not superiority as the EMPA-REG OUTCOME trial. The results of the EMPA-REG OUTCOME study were achieved in a cohort with long-standing diabetes and cardiovascular disease and may not be automatically generalised for patients with new onset type 2 diabetes before having developed concomitant vascular complications. Here, metformin is still standard and has proved to be advantageous. The long-term effects of SGLT-2 inhibitors and empagliflozin over >10 years are still unknown and have to be established in comparison to classic antidiabetic drugs. Adverse events and safety characteristics are of utmost importance in medications for chronic diseases. In this respect, the rare reports on ketoacidotic metabolic disturbances that have been associated with the use of SGLT-2 inhibitors have to be taken into account as well. The respective cardiovascular safety study results with the other available SGLT-2 inhibitors canagliflozin and dapagliflozin have also to be completed in order to confirm these data and in order to have a broader picture of the action of the drug class of SGLT-2 inhibitors. At present, there is an indication to treat patients with type 2 diabetes and a similar cardiovascular risk profile as seen in the study population of the EMPA-REG Outcome trial with empagliflozin. In addition, there are now stimulating and positive data to explore the action of SGLT-2 inhibitors further in order to explain the findings of the EMPA-REG Outcome study in more detail and in mechanistic pathophysiological models. This may help to evolve the drug class further and may help to identify patient groups that have a specifically high benefit from such a therapy.^7^
